# Effect of self-monitoring on long-term patient engagement with mobile health applications

**DOI:** 10.1371/journal.pone.0201166

**Published:** 2018-07-26

**Authors:** Kyunghee Lee, Hyeyon Kwon, Byungtae Lee, Guna Lee, Jae Ho Lee, Yu Rang Park, Soo-Yong Shin

**Affiliations:** 1 Mike Ilitch School of Business, Wayne State University, Detroit, Michigan, United States of America; 2 Department of Biomedical Informatics, Asan Medical Center, Seoul, Korea; 3 KAIST College of Business, Seoul, Korea; 4 Division of Nursing Science, College of Health Science, Ewha Womans University, Seoul, Korea; 5 Department of Emergency Medicine, Asan Medical Center, University of Ulsan College of Medicine, Seoul, Korea; 6 Department of Biomedical Systems Informatics Yonsei University College of Medicine, Seoul, Korea; 7 Department of Digital Health, SAIHST, Sungkyunkwan University, Seoul, Korea; National Cancer Institute, UNITED STATES

## Abstract

Despite the growing adoption of the mobile health (mHealth) applications (apps), few studies address concerns with low retention rates. This study aimed to investigate how the usage patterns of mHealth app functions affect user retention. We collected individual usage logs for 1,439 users of single tethered personal health record app, which spanned an 18-months period from August 2011 to January 2013. The user logs contained timestamps whenever an individual uses each function, which enables us to identify the usage patterns based on the intensity of using a particular function in the app. We then estimated how these patterns were related to 1) the app usage over time (using the random effect model) and 2) the probability of stopping the use of the application (using the Cox proportional hazard model). The analyses suggested that the users utilize the app most at the time of the adoption and gradually reduce their usage over time. The average duration of use after starting the app was 25.62 weeks (SD: 18.41). The degree of the usage reduction, however, decreases as the self-monitoring function is more frequently used (coefficient = 0.002, P = 0.013); none of the other functions has this effect. Moreover, engaging with the self-monitoring function frequently (coefficient = −0.18, P = 0.003) and regularly (coefficient = 0.10, P = 0.001) significantly also reduces the probability of abandoning the application. Specifically, the estimated survival rate indicates that, after 40 weeks since the adoption, the probability of the regular users of self-monitoring to stay in use was about 80% while that of non-user was about 60%. This study provides the empirical evidence that sustained use of mHealth app is closely linked to the regular usage on self-monitoring function. The implications can be extended to the education of users and physicians to produce better outcomes as well as application development for effective user interfaces.

## Introduction

Personal health records (PHRs) have rapidly become renowned for their capabilities to support patient-centered care. They enable patients to be involved in care processes by granting them access to and control over their health information [1–3], which results in better patient satisfaction [4,5], and improvement in care services [6] and clinical outcomes [7]. The advance in mobile technology has further strengthened such advantages of PHRs; the use of mobile PHR (mPHR) applications (apps) enables patients to access, monitor, record, and update their health information regardless of physical constraints such as time and location [8].

Researchers have started to investigate the benefits of the new technology from diverse perspectives and shown fruitful outcomes. The majority of these studies have been dedicated to adoption-stage research, which includes the usability, feasibility, and acceptability of mobile-enabled care systems, by both patients [9,10] and care professionals [11,12]. Another stream of studies has focused on its capability to enhance patient adherence to treatment [13] and, therefore, improve the clinical outcomes of chronic diseases such as diabetes mellitus [14] and cardiovascular diseases [15]. Although the debates have yet to converge to a decisive conclusion, the new systems have shown potential to transform and advance the current healthcare system to a better form [16].

However, a critical problem remains unsolved: user retention. According to recent statistics, more than two-thirds of people who downloaded a mobile health (mHealth) app used it only once and stopped using it [17]. The low retention rate is a critical issue given the nature of chronic diseases, for which most mHealth apps are designed. Changing habitual behavior, which is a predominant factor in preventing premature death [18], requires a person to devote a substantial amount of time. To benefit from this new technology, therefore, users must use the apps for a sufficient period to incorporate them into their daily lives [19,20]. Despite the importance of mHealth apps, minimal attention has been drawn to how patients engage in their sustained use [21,22]. Although mHealth promoters have studied its adaptability, few studies have distinguished the concept of sustained use from initial adoption [23–25]. This paucity of research hinders care providers from getting relevant implications for patient engagement with mHealth apps [26].

The low retention rate could stem from the incorrect use of the applications in a way to realize benefits from the technology adoptions. The technology adoption literature suggested that, while initial adoption of technology is related to the cost of exploring an innovation such as perceptions of compatibility or visibility, long-term engagement is primarily driven by rational considerations, including the benefits offered by the innovation (relative advantage) and the ability of users to recognize these benefits (result demonstrability) [27]. Thus, users may become attracted to the availability of new technology. However, this attractiveness maybe short-lived unless they develop patterns or practices of using this technology to obtain its relative benefits over past routines.

This study investigated how the usage patterns of PHR apps functions affect user retention. Specifically, people use PHR apps differently in intensively utilizing a particular feature among many others available in the app, such as functions for self-monitoring users’ health data, access to electronic medical records (EMRs) stored in hospitals, medication management, and appointment reservations. With a detailed usage log of an mPHR app, we identified the distinctive patterns that users engaged with the various app functions, measured differences in the app usage levels and probability to stop using the app.

## Methods

### Data description

We collected log data of an mPHR app, termed “My Chart in My Hand” (MCMH), which was developed by Asan Medical Center (AMC) in Korea [28]. MCMH was the first mPHR app launched in Korea on December 2010 and upgraded to MCMH version 2 in January 2016. The development of this MCMH was started in February 2010 to assist patients in checking and managing their own health records. The app was developed by AMC with the collaboration of a telecommunication company in Korea. With a simple online registration, anyone who had visited AMC could check their hospital records and manage their health information via the app for free. MCMH provides four functions to aid users to self-manage their health. First, the self-monitoring function provides features for tracking and updating PHRs such as blood glucose levels, blood pressure levels, weight, and height. Users are required to manually input data; there is no functionality of accepting data streams from personal tracking devices. Based on the user inputs, the app provides them with some useful indexes such as the body mass index, 10-year cardiovascular disease risk, and metabolic syndrome risk. Second, in the chart function, patients can access their medical records, which are stored in the hospital, and manage their disease and allergy history. Third, the medication function provides patients with medication schedules and information about medicines. At last, patients can make appointments and check their waiting status using the outpatient support service. Note that the users voluntarily adopted this app; physicians had never tried to encourage or convince patients to adopt the app during the session. Moreover, though MCMH was not designed for research purpose, the usage monitoring system which shows a user log was, developed in August 2011 to improve the app’s functionality.

We collected individual usage logs for 1,439 users, which spanned 18 months from August 2011 to January 2013 with the approval of the Institutional Review Board of AMC. The usage log was collected using the usage monitoring system. The user logs contained time stamps for each function, which are recorded whenever an individual uses these functions. We also gathered demographics and medical records for these users, such as age, gender and the number of outpatient visits, admissions, and ER visits. It also included patient’s diagnosis and medication information, coded by the ICD-10 (International Classification of Diseases, 10^th^ version) and ATC (Anatomical Therapeutic Chemical Classification System) codes, respectively. These variables are time-invariant and were collected once for the entire sampling period.

This study was approved by the institutional review board of the hospital (IRB no. 2013–0102). The need for informed consent was waived by the ethics committee, as this study utilized routinely collected log data that were anonymously managed at all stages, including during data cleaning and statistical analyses.

### Statistical method

We analyzed the log data using two different approaches: the random-effect model to observe changes in the app usage levels over time and the Cox proportional hazard model to observe changes in the probability of stopping using the app.

#### Random-effect model

The usage-level random-effect model is developed as follows:

$$\begin{array}{l} LOG\_IN_{i,t}=\beta_{1}+\beta_{2}ELAPSED_{i,t}+\beta_{3}SM_{i,t-1}+\beta_{4}CHART_{i,t-1}+\beta_{5}MED_{i,t-1}+\beta_{6}OSS_{i,t-1}\\ \;+\beta_{7}SM_{i,t-1}\times ELAPSED_{i,t}+\beta_{8}CHART_{i,t-1}\times ELAPSED_{i,t}\\ \;+\beta_{9}MED_{i,t-1}\times ELAPSED_{i,t}+\beta_{10}OSS_{i,t-1}\times ELAPSED_{i,t}\\ \;+Χ\delta +\varepsilon_{i,t}\\ \;\varepsilon_{i,t}=\nu_{i}+\eta_{i,t} \end{array}$$ (1)

The dependent variable LOG_IN is the number of times patient *i* logged in the app in a given week *t*. ELAPSED represents the number of weeks since patient *i* adopted MCMH. Thus, the coefficient *β*_2_ captures the user bounce rate over time: the changes in the number of log-ins along with the length of use. If *β*_2_ is negative, then it presumably indicates users reduce log-ins as they use the app longer, and *vice versa*.

The frequencies of use of the app functions namely, self-monitoring (SM), chart (CHART), medication (MED), and outpatient support service (OSS) were included in the model. Note that the frequency variables were lagged (t-1) while the dependent variable used the current time (t). This time gap enables exclusion of the possibility of a reverse relationship from logins to individual function uses and, thus, can support causality of our findings. We further interacted the frequency of function usage with the elapsed weeks (ELAPSED) to examine how the bound rate varies across users depending on their usage patterns in terms of the frequency of function usage. The coefficients, *β*_7−10_, thus captured the moderating effects of the function usage on the degree of decreasing usage over time. The control variables **X** included patient’s age, gender, number of outpatient visits, number of hospital admissions, number of emergency room visits, and disease type.

In the random-effect model, the error, ε_*i,t*_ consisted of two components–unobserved patient-specific characteristics, *v_i_*, and disturbances, *η_i,t_*. The former component is to control for individual heterogeneity (See S3 Table for details about the methods).

It is worth noting that when the interaction terms (e.g., ELAPSED*SM) were included in the model, the component terms of each function represented conditional effects, as if the value of the elapsed weeks is zero, which means the first week of the adoption. Hence, the coefficients of the component terms, *β*_3−6_, indicate the immediate *short-term* effects at the point of adoption, while those of the interaction terms, *β*_7−10_, measure the *long-term* effects as time passes.

#### Survival analysis

For the survival analysis, we transformed the raw data into a cross-sectional form and employed the Cox proportional hazard function [29] to predict which pattern of function usage is associated with the likelihood of stopping using the app. To capture the patterns, frequency and regularity of function usage were considered the average of the usage of the functions (e.g., AVG_xxx) for the frequency and the standard deviation of the usage of the functions (e.g., STD_xxx) for the regularity. To control for the individual heterogeneity in app usage in general, we also included the average of number of days used per week (AVG_DAY) and its standard deviation (STD_DAY). The time-invariant control variables X were also equally included as in the random-effect model.

Identifying the discontinuing event for the survival analysis was challenging due to the nature of the event. Unlike diagnosable diseases or symptoms which could be immediately identified at a given time point, discontinuing the use of the app required a period of observation to determine. For example, a patient could be considered a drop-out user when she/he had not used the app at all for the last few months before the end of the observation period. Consequently, a period was chosen for making this decision. In particular, a user was considered to have no longer used the app if he/she had not logged-in for the last month (i.e., December 31 –January 31); otherwise, the user was considered to have maintained use. For robustness, we also considered two alternative periods: three months (October 30 –January 31) and five months (August 31 –January 31) before termination. We only presented the results of the one-month period for brevity since all results from these three choices showed no qualitative differences. The results of all three periods are reported in S3 Table.

## Results

The usage log and demographic data and medical records for 1,439 patients were analyzed. The descriptions and statistics of these variables are listed in S1 and S2 Tables.

### Distribution of function usage

**Fig 1:**
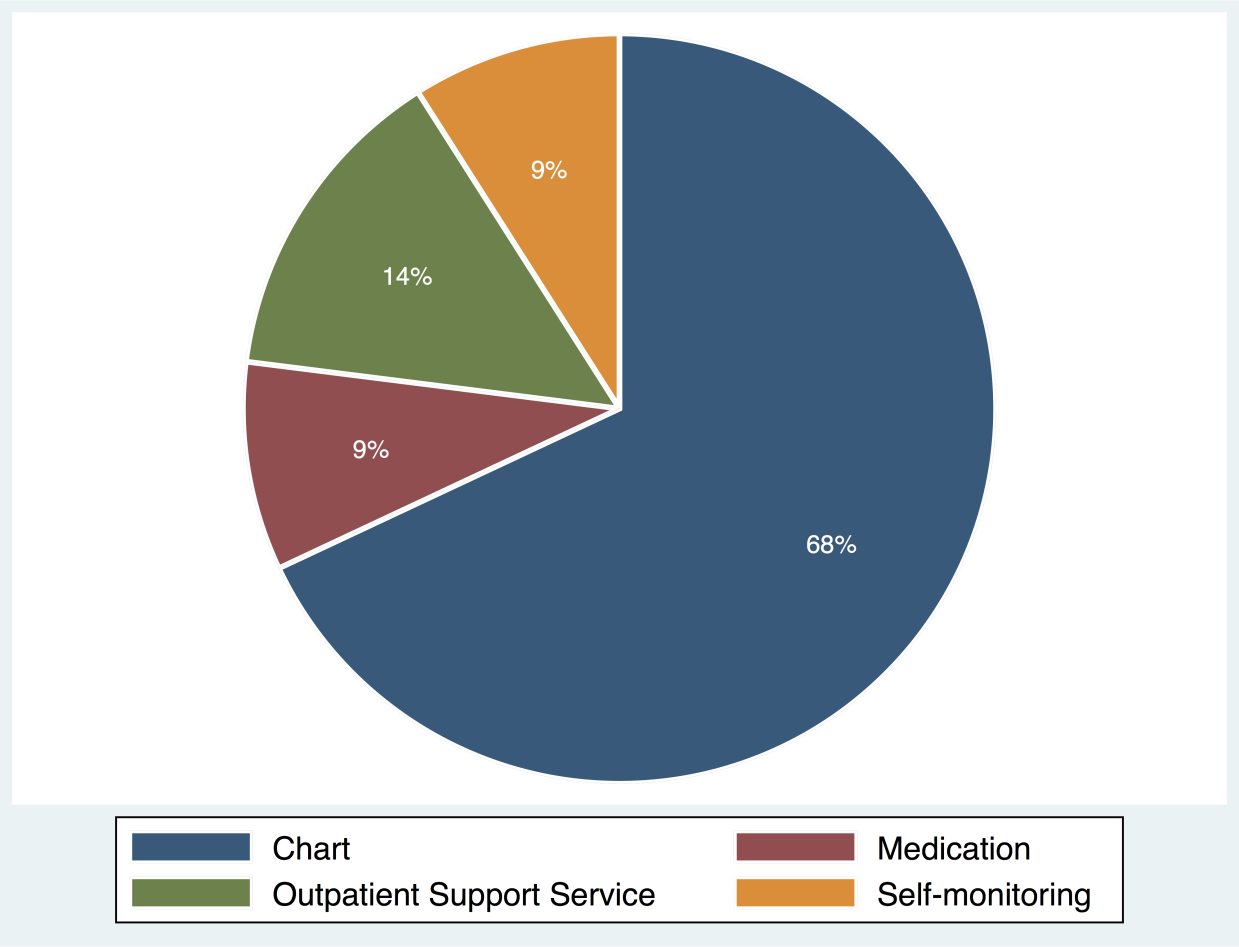
Distribution of usage of each function in MCMH.

Fig 1 illustrates the distribution of usage of each function in MCMH. Users used the app mostly for acquiring EMRs with the chart function; the average of weekly usage of the chart function is 5.06 and accounts for 68% of the total usage. The outpatient support service function was used 0.96 per week on average and accounts for 14% of the total usage. The medication function was used 0.55 on average and the self-monitoring function was 0.49, each accounting for approximately 9% of the total usage. This distribution clearly demonstrated that there were distinctive patterns of function usage in the app, and the chart function occupied most usage while the self-monitoring accounted for just a fraction.

### Results of the random-effect model: Change in usage level over time

The results of the random-effect model are listed in Table 1. The estimation model is Eq. (1) with the number of log-ins as the dependent variable. Note that we employed clustered standard errors to correct for possible correlation of errors within a cluster, or user [30].

**Table 1:** The impacts of different mPHR functions on patient’s app usage.

		Number of log-ins	
		Coefficient	*P*-value
Elapsed weeks since adoption (ELAPSED)		-0.0059	0.000
Number of self-monitoring usage (SM)		0.0247	0.025
Number of chart usage (CHART)		0.0517	0.000
Number of medication usage (MED)		0.0089	0.389
Number of outpatient support service usage (OSS)		0.0251	0.003
Interaction term^a^			
	SM × ELAPSED	0.0020	0.013
	CHART × ELAPSED	0.0001	0.929
	MED × ELAPSED	-0.0002	0.677
	OSS × ELAPSED	-0.0002	0.636
Control variables^b^		Included	

Note

^a^ The interaction terms measure how the coefficient of ELAPSED is different for different usages of each function (SM, CHART, MED, and OSS).

^b^The coefficients of the control variables were not reported for brevity. The control variables included patient’s age, gender, number of outpatient visits, number of hospital admissions, number of emergency room visits, and disease type (See S1 Table for details).

In Table 1, the negative coefficient of the elapsed weeks (ELAPSED; -0.0059, P < 0.05) indicates the declining app usage over time; users reduced their usage of the app as time passed since the initial adoption. The degree of the reduction, however, varied with which function they had used. The interaction between the frequency of the self-monitoring usage and number of elapsed weeks had a positive and significant coefficient, which indicates that the degree of the usage reduction decreases as the self-monitoring function is more frequently used (SM × ELAPSED; 0.0020, P < 0.05); the self-monitoring function was to flatten the negative slope of the app usage.

The other functions (CHART, MED, and OSS), however, exhibited different regularities. The medication and outpatient support service functions had no statistically significant impact on preventing users from reducing their app usage over time (MED × ELAPSED and OSS × ELAPSED were insignificant).

As mentioned, the component term indicated short-term effects at the point of adoption while the interaction term long-term effects as time passed. Thus, the positive coefficients of the component terms, CHART and OSS indicates positive short-term effects, but they diminish quickly based on the insignificant interaction terms (CHART × ELAPSED and OSS × ELAPSED).

### Results of the survival analysis: Probability of stopping using app

Table 2 lists the distribution of users with respect to abandonment or use of the mPHR app. Assuming users who did not use the app for more than one month since the last use as lost users, 44% of the total users had abandoned the app.

**Table 2:** Distribution of inactive users of the mPHR app.

Unused period	One-month	
	Number of users	%
Abandon	674	46%
Use	765	54%
Total	1439	100%

Table 3 reports the results of the survival analysis. In Table 3, the coefficient of the average weekly usage of the self-monitoring (AVG_SM; -0.18, P < 0.01) was negative and significant, and the coefficient of its standard deviation (STD_SM; 0.10, P < 0.01) was positive and significant. This suggests that frequent and regular use of the self-monitoring function increased the chance of users continuing to use the app. On the contrary, the frequent and regular use of the other functions decreased the chance.

**Table 3:** The impacts of different mPHR functions on the probability of users abandoning the app.

	Likelihood of abandonment	
	Coefficient	P-value
Average usage of self-monitoring function of patient *i* per week (AVG_SM)	-0.18	0.003
Average usage of chart function of patient *i* per week (AVG_CHART)	0.02	0.374
Average usage of medication function of patient *i* per week (AVG_MED)	0.10	0.044
Average usage of outpatient support service of patient *i* per week (AVG_OSS)	0.13	0.000
Standard deviation of usage of self-monitoring function of patient *i* per week (STD_SM)	0.10	0.001
Standard deviation of usage of chart function of patient *i* per week (STD_CHART)	-0.04	0.052
Standard deviation of usage of medication function of patient *i* per week (STD_MED)	-0.09	0.065
Standard deviation of usage of outpatient support service of patient *i* per week (STD_OSS)	-0.05	0.008

Note: The coefficients of the control variables were not reported for brevity. The control variables included patient’s age, gender, number of outpatient visits, number of hospital admissions, number of emergency room visits, disease type, and individual heterogeneity in app usage such as the average and standard deviation of the number of days used per week (See S4 Table for details).

The unused period was one month for this result. See S3 Table for the results of all three periods (one, three, five-month)

**Fig 2:**
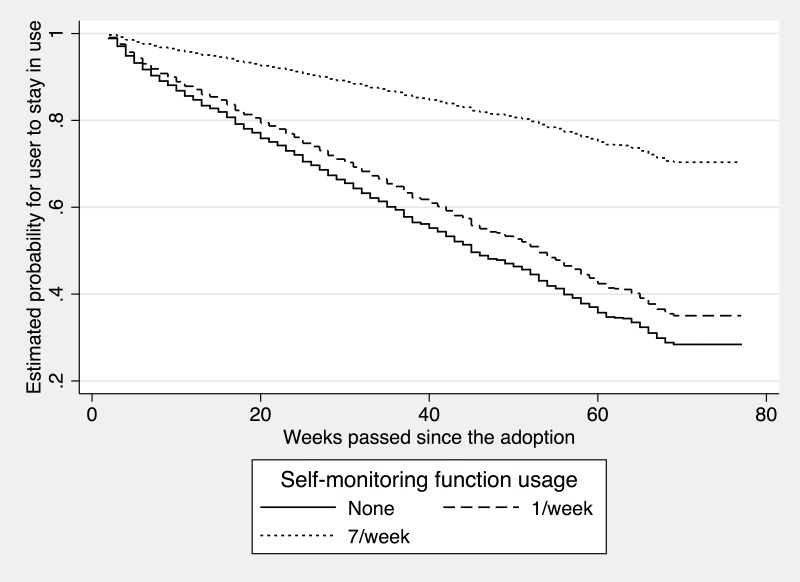
Effects of the self-monitoring.

Fig 2 visualizes the effects of the self-monitoring. Specifically, the probability of continuing using the app on the y-axis is computed by inserting the different levels of usage of the self-monitoring function (i.e., none, once a week, and seven times a week) into the estimation models. In Fig 2, the likelihood of continued use decreased over time for all three lines, but the slopes varied depending on the intensity of the self-monitoring use; the slope of the line at the top, which represents a user who used the self-monitoring function seven times a week, is flatter than that of the other two representing users who used the function less frequently. The estimated survival rate suggests that, after 40 weeks since the adoption, the probability of the regular users of the function (i.e., seven times a week) maintaining app use was about 80% while that of the non-user of the function was about 60%.

To understand the differences between demographic groups, we also run subsample analyses by two dimensions–gender (male vs. female) and age (age < 40 vs. age ≥ 40). Previous research demonstrated that gender, age, and education affects adoption of the mHealth app [31]. Across the gender and age comparisons, the moderating effects of the self-monitoring feature are stronger for those who are presumably considered more technically inclined (the male group and the younger group) than for those who are not (the female group and the elder group). This indicates that individuals' technical competence is an important factor in mHealth adoption behaviors, as in the previous research. The detailed results can be found in S5 Table.

## Discussion

This study investigated whether users differently adopt functions in mPHR apps and how the distinctive patterns of function usage affect their retention rate. To gain empirical insight, a large-scale usage log of a mPHR app was collected and analyzed using econometric methodology.

Consistent with the anecdotal evidence, we first found that users significantly reduced their usage levels over time; the number of log-ins decreased in the weeks that elapsed since adoption. The usage reduction can be explained from several perspectives. First, the results were consistent with the satiation effect in which people are less likely to sustain current usage because their marginal utility, which was obtained from the use of the app, diminished with consumption [32]. Second, from a psychophysiological point of view, this result may stem from desensitization. For instance, greater use of the app tends to desensitize patients to alarmist health information from the app, which produces low levels of usage. In either explanation, the finding provided evidence that people tended to reduce their usage over time and eventually stopped using the mPHR app. The declining trend justifies the need to promote the sustained use of the app; otherwise, all expenses spent for the development and distribution of mPHR apps are futile.

As a solution to this concern, our study provides a theoretical foundation and empirical evidence for how patients become committed to the sustained use of mHealth apps via the self-monitoring mechanism, i.e., entering and monitoring users’ health data. Involved in the mechanism, users are motivated to track, record, and update their health information and therefore be accurately informed about the consequences of their daily activities, which can lead to healthier behavior and better clinical outcomes. Such potential benefits, therefore, motivate them to utilize the technology longer. Our findings suggest that the negative slope of the declining trends in app usage was flattened by the use of the frequent self-monitoring function, which supports a positive long-term effect of the self-monitoring. These findings suggested an important implication: users obtain different levels of utility depending on which function they intensively utilize. Frequent use of the functions not providing clear benefits may increase only the satiation effect that users obtain less utility over consumption and thus renders the decision to not use the app. mHealth apps therefore should be designed in a way to engage users in the right functions to stimulate behavioral changes, which is key to promoting long-term engagement.

This study also presents evidence that although most people used the app mainly for the chart function, the committed users of the app were those who used the self-monitoring function. In other words, the function that attracted people to use the app more was different from the one that made them use it consistently. According to the distribution of function usage in our data, the users appeared to focus on acquiring EMR information from the chart function (68%) and making reservations using the outpatient support service (14%); however, they spend less time on the self-monitoring (9%) and medication (9%) functions. This asymmetric distribution suggests that users’ primary purpose for using the app was to acquire their medical information with the chart function, and the self-monitoring function was not their main interest. Nevertheless, this small fraction of the usage turns out to be much more effective in promoting the sustained use than any other function of this app. In fact, our findings suggest that only the users committed to the self-monitoring were willing to continue using the app; the other functions were ineffective or, even worse, harmful to the long-term engagement. Thus, it is crucial to understand that each app function has unique impacts on user behavior and how to utilize the differences to achieve diverse goals, which is key to further advancing mHealth apps.

Our study has several limitations. First, there may have been patient-specific heterogeneity. For example, a history of the family disease, a critical factor of chronic diseases, might influence patients’ attitude toward mPHR apps and usage pattern because they are more likely to be cautious about their health conditions. We attempted to address this issue by including demographic factors, hospital records, and disease types for each individual and employing a random-effect model to consider patient-specific components. Second, the definition of abandonment was based on prediction instead of observed facts. Because our data were right-censored, we were unable to observe whether abandonment was made differently. Although censored data are common in survival analysis and we attempted to alleviate this concern by applying the different definitions of abandonment, it might decrease the predictability of our models. Third, our sample might represent early adopter groups because MCMH was the first mPHR app and our data were collected at an early stage (18 months since the app was introduced), which could limit the generalizability of our findings. The comparison of the hospital record variables between the user and non-user groups revealed that the users visited the hospital more often (17.11) than the non-users (5.49). However, this concern is only valid when an unobservable characteristic that only existed in the user group simultaneously affected their usage of the functions (i.e., explanatory variables) and long-term engagement (i.e., dependent variables). We also attempted to minimize the concern by adding a set of control variables, including demographic information, hospital records, and diseases types and employing econometric methods. Nevertheless, it is nearly impossible to completely eliminate such concerns in empirical studies. Thus, the findings should be cautiously interpreted prior to applying them to disease-specific apps. Fourth, Koreans are well-known for high technology adoption rate. This could be another limitation of this study. Follow-up research should be performed in other countries.

The development of mHealth apps remains at an early stage, which calls for research to reveal essential elements for reaching its full potential. This study contributes to the literature by providing theoretical reasoning and empirical evidence that mHealth app users are more likely to continue using the app when committed to the self-monitoring mechanism. This finding leads to a prominent implication: having users engage with a proper function in mPHR apps is critical to encourage them to use the app longer. The efficacy of the self-monitoring validated in this study represents a potential method for encouraging behavioral changes in patients and provides insights into the design of mHealth apps. As a future work, classifying different types of users is necessary to improve long-term engagement with an mHealth app [33], since the users who want to be more involved with their health are more motivated to use the app and the self-monitoring function helps feed into this motivation.

## Supporting information

S1 Table Variable descriptions and summary statistics. (DOCX)

S2 Table Age distribution. (DOCX)

S3 Table Distribution of inactive users of the mPHR app. (DOCX)

S4 Table The impacts of different mPHR functions on the probability of users abandoning the app. (DOCX)

S5 Table The difference between demographic groups. (DOCX)
