# Supplementary material for: Effect of self-monitoring on long-term patient engagement with mobile health applications
Source: PLoS One. 2018 Jul 26;13(7):e0201166. doi: 10.1371/journal.pone.0201166 (PMC6062090; doi:10.1371/journal.pone.0201166)
Supplement: S1 Table — (DOCX) [file pone.0201166.s001.docx]

**S1 Table****. Variable descriptions and summary statistics.**

| **Variable** | | | **Mean** | **Std** | **Min** | **Max** |
| --- | --- | --- | --- | --- | --- | --- |
| 1. Panel data (*i, t*) | | | | | |  |
|  | Number of logins by patient *i* in week *t* (LOG_IN) | 1.40 | 4.40 | 0 | 294 |  |
|  | Number of self-monitoring function usage by patient *i* in week *t* (SM) | 0.49 | 4.37 | 0 | 401 |  |
|  | Number of chart function usage by patient *i* in week *t* (CHART) | 5.06 | 19.33 | 0 | 610 |  |
|  | Number of medication function usage by patient *i* in week *t* (MED) | 0.55 | 4.93 | 0 | 642 |  |
|  | Number of outpatient support service usage by patient *i* in week *t* (OSS) | 0.96 | 4.21 | 0 | 227 |  |
|  | Elapsed weeks since app adoption by patient *i* (ELAPSED) | 25.62 | 18.41 | 1 | 75 |  |
| 2. Cross-sectional data (*i*) | | | | | |  |
|  | Average usage of self-monitoring function by patient *i* per week (AVG_SM) | 0.83 | 3.77 | 0 | 106 |  |
|  | Average usage of chart function by patient *i* per week (AVG_CHART) | 6.50 | 14.32 | 0 | 251 |  |
|  | Average usage of medication function by patient *i* per week (AVG_MED) | 0.81 | 1.88 | 0 | 43.73 |  |
|  | Average usage of outpatient support service by patient *i* per week (AVG_OSS) | 1.29 | 2.38 | 0 | 40.50 |  |
|  | Standard deviation of usage of self-monitoring function by patient *i* per week (STD_SM) | 1.76 | 4.50 | 0 | 73.53 |  |
|  | Standard deviation of usage of chart function by patient *i* per week (STD_CHART) | 9.41 | 14.62 | 0 | 223.45 |  |
|  | Standard deviation of usage of medication function by patient *i* per week (STD_MED) | 1.94 | 3.96 | 0 | 87.37 |  |
|  | Standard deviation of usage of outpatient support service by patient *i* per week (STD_OSS) | 2.50 | 3.52 | 0 | 52.34 |  |
| 3. Control variables (*i*): Time invariant and employed in both panel analysis and survival analysis | | | | | |  |
|  | Age of patient *i* (AGE)^a^ | 3.21 | 1.66 | 0 | 7 |  |
|  | Gender of patient *i* (GENDER) | 0.55 | 0.50 | 0 | 1 |  |
|  | Number of outpatient visits of patient *i* (OPD) | 17.31 | 15.10 | 0 | 65 |  |
|  | Number of hospital admissions of patient *i* (ADM) | 2.38 | 4.24 | 0 | 43 |  |
|  | Number of emergency room visits of patient *i* (ER) | 1.22 | 2.64 | 0 | 28 |  |
| 4. Additional control variables (*i*) for survival analysis | | | | | |  |
|  | Average of number of days that patient *i* used the app in a week (AVG_DAY) | 0.80 | 0.87 | 0.03 | 6.18 |  |
|  | Standard deviation of the number of days that patient i used the app in a week (STD_DAY) | 0.84 | 0.55 | 0 | 4.24 |  |

^a^Age of patient is categorized into one-digit numbers (0: 0–9, 1: 10–19, 2: 20–29, 3: 30–39, 4: 40–49, 5: 50–59, 6: 60–69, and 7: 70–79)

^b^1 for male; 0 for female
