# Supplementary material for: Effect of self-monitoring on long-term patient engagement with mobile health applications
Source: PLoS One. 2018 Jul 26;13(7):e0201166. doi: 10.1371/journal.pone.0201166 (PMC6062090; doi:10.1371/journal.pone.0201166)
Supplement: S2 Table — (DOCX) [file pone.0201166.s002.docx]

**S2 Table. Age distribution.**

| **Age** | **Female (%)** | | **Male (%)** | | **Total** |
| --- | --- | --- | --- | --- | --- |
| 20s | 169 | (64%) | 96 | (36%) | 265 |
| 30s | 248 | (52%) | 227 | (48%) | 475 |
| 40s | 92 | (28%) | 236 | (72%) | 328 |
| 50s | 95 | (41%) | 134 | (59%) | 229 |
| 60s | 37 | (37%) | 64 | (63%) | 101 |
| 70s ~ | 12 | (29%) | 29 | (71%) | 41 |
| Total | 653 | (45%) | 786 | (55%) | 1439 |
