# Supplementary material for: Effect of self-monitoring on long-term patient engagement with mobile health applications
Source: PLoS One. 2018 Jul 26;13(7):e0201166. doi: 10.1371/journal.pone.0201166 (PMC6062090; doi:10.1371/journal.pone.0201166)
Supplement: S3 Table — (DOCX) [file pone.0201166.s003.docx]

**S3 Table.** **Distribution of inactive users of the mPHR app.**

| **Time period** | **Five months** | | **Three months** | | **One month** | |
| --- | --- | --- | --- | --- | --- | --- |
|  | **Number of users** | **%** | **Number of users** | **%** | **Number of users** | **%** |
| **Abandon** | 381 | 22% | 539 | 32% | 739 | 44% |
| **Use** | 1313 | 78% | 1155 | 68% | 955 | 56% |
| **Total** | 1694 |  | 1694 |  | 1694 |  |
