# Supplementary material for: Effect of self-monitoring on long-term patient engagement with mobile health applications
Source: PLoS One. 2018 Jul 26;13(7):e0201166. doi: 10.1371/journal.pone.0201166 (PMC6062090; doi:10.1371/journal.pone.0201166)
Supplement: S4 Table — (DOCX) [file pone.0201166.s004.docx]

**S4 Table.** **The impacts of different mPHR functions on the probability of users abandoning the app.**

|  | **Five months** | | **Three months** | | **One month** | |
| --- | --- | --- | --- | --- | --- | --- |
|  | **Coefficient** | **P-value** | **Coefficient** | **P-value** | **Coefficient** | **P-value** |
| **Average of weekly usage of self-monitoring function (AVG_SM)** | −0.13 | 0.009 | −0.11 | 0.013 | −0.11 | 0.009 |
| **Average of weekly usage of chart function (AVG_CHART)** | 0.03 | 0.148 | 0.04 | 0.011 | 0.03 | 0.125 |
| **Average of weekly usage of medication function (AVG_MED)** | 0.21 | 0.007 | 0.15 | 0.008 | 0.15 | 0.003 |
| **Average of weekly usage of outpatient support service (AVG_OSS)** | 0.14 | 0.000 | 0.14 | 0.000 | 0.14 | 0.000 |
| **Standard deviation of weekly usage of self-monitoring function (STD_SM)** | 0.08 | 0.006 | 0.07 | 0.014 | 0.07 | 0.005 |
| **Standard deviation of weekly usage of chart function (STD_CHART)** | −0.06 | 0.007 | −0.06 | 0.000 | −0.04 | 0.019 |
| **Standard deviation of weekly usage of MED (STD_MED)** | −0.19 | 0.037 | −0.11 | 0.071 | −0.11 | 0.02 |
| **Standard deviation of weekly usage of outpatient support service (STD_OSS)** | −0.06 | 0.010 | −0.07 | 0.000 | −0.07 | 0.000 |
| **Control variables** | | | | | | |
| **Average of weekly usage of app (AVG_USE)** | 0.74 | 0.001 | 0.64 | 0.001 | 0.65 | 0.001 |
| **Standard deviation of weekly usage of app (STD_USE)** | −0.58 | 0.095 | −0.70 | 0.022 | −0.73 | 0.011 |
