# Supplementary material for: Effect of self-monitoring on long-term patient engagement with mobile health applications
Source: PLoS One. 2018 Jul 26;13(7):e0201166. doi: 10.1371/journal.pone.0201166 (PMC6062090; doi:10.1371/journal.pone.0201166)
Supplement: S5 Table — (DOCX) [file pone.0201166.s005.docx]

**S5 Table.** **The difference between demographic groups.**

| **DV: Login** | **Gender** | | **Age** | |
| --- | --- | --- | --- | --- |
|  | **Male** | **Female** | **Below 40** | **Above 40** |
| ELAPSED | −0.0067^***^  (0.000) | −0.0046^***^  (0.010) | −0.0065^***^  (0.000) | −0.0050^***^  (0.000) |
| SM × ELAPSED | 0.0027^***^  (0.008) | 0.0012  (0.291) | 0.0023^*^  (0.089) | 0.0014^***^  (0.005) |
| CHART × ELAPSED | 0.0005  (0.579) | −0.0006  (0.314) | 0.0009  (0.381) | −0.0006  (0.192) |
| MED × ELAPSED | −0.0005  (0.420) | −0.0006  (0.355) | −0.0007  (0.351) | −0.0000  (0.984) |
| OSS × ELAPSED | −0.0008  (0.189) | 0.0005  (0.160) | −0.0006  (0.353) | 0.0001  (0.761) |

Note: The interaction terms measure how the coefficient of ELAPSED is different for different usages of each function (SM, CHART, MED, and OSS).

The coefficients of the control variables were not reported for brevity. The control variables included patient’s age, gender, number of outpatient visits, number of hospital admissions, number of emergency room visits, and disease type (See S1 Table for details). p-values in parentheses. * p < 0.10, ** p < 0.05, *** p < 0.01
